# Biomedical Properties, Developments and Therapeutic Potential of Sesquiterpenoid Lactones and Natural Compounds

**DOI:** 10.3390/ph19081318

**Published:** 2026-08-20

**Authors:** Normand García-Hernández, Israel Ramírez-Sánchez, Rosa María Ordoñez-Razo

**Affiliations:** 1 División de Innovación y Regulación de la Investigación en Salud, Instituto Mexicano del Seguro Social, Av. Cuauhtémoc 330, Col. Doctores, Ciudad de México 06720, Mexico; normand.garcia@imss.gob.mx; 2 Sección de Estudios de Posgrado e Investigación, Escuela Superior de Medicina, Instituto Politécnico Nacional, Plan de San Luis y Díaz Mirón s/n, Col. Casco de Santo Tomás, Ciudad de México 11340, Mexico; israel.ramirez14@hotmail.com; 3 Unidad de Investigación Médica en Genética Humana, Hospital de Pediatría “Dr. Silvestre Frenk Freund”, Centro Médico Nacional Siglo XXI, Instituto Mexicano del Seguro Social, Av. Cuauhtémoc 330, Col. Doctores, Ciudad de México 06720, Mexico

The study of plant-derived secondary metabolites has advanced the field of pharmacochemistry. Natural products are a rich source of potential therapeutics. These compounds can benefit patients and healthcare systems by offering innovative, targeted treatments for various diseases.

This Special Issue, “Biomedical Properties, Developments and Therapeutic Potential of Sesquiterpenoid Lactones and Natural Compounds,” features original research highlighting the innovative aspects of sesquiterpene lactones and other natural compounds in the development of new pharmacological strategies. These strategies apply to oncology, infectious diseases, wound healing, and nanotechnology-enhanced therapeutics. Collectively, the proposed approaches aim to overcome treatment challenges and accelerate the translation of laboratory findings to clinical application.

In this context, sesquiterpene lactones are among the most important families of natural compounds. They are secondary metabolites mainly obtained from plants of the Asteraceae family [1,2]. These are highly interesting due to their anti-inflammatory, cytotoxic, antibacterial, antiprotozoal, antiviral, and antineoplastic properties [3,4,5,6]. Thus, they play an important role in developing new treatments. This issue presents two contributions that analyze the sesquiterpene lactone called Incomptine A (IA) as a therapeutic candidate for Non-Hodgkin Lymphoma (NHL) in a mouse model. To explore the effect of IA treatment, more than 2700 proteins were identified using proteomics mass spectrometry. Of these, 412 were significantly altered by IA treatment. The modified proteins are involved in transcriptional regulation, chromatin remodeling, oxidative phosphorylation, cytoskeletal organization, and necroptosis. The Fhl1 protein was proposed as a potential therapeutic target and prognostic biomarker for NHL. Further pathway analyses revealed effects in glycolysis, epigenetic regulation, and immune-related signaling. Docking studies also supported the IA pharmacokinetics [7,8]. Taken together, these findings suggest that IA is a multi-target natural metabolite that can cause cell death and disrupt metabolism in malignant lymphocytes. However, detailed studies are still required to validate the protein targets, verify changes in protein levels in lymph nodes, clarify how specific proteins altered by IA are involved in NHL, and demonstrate that IA could be an effective therapy for NHL.

**Natural and repurposed compounds as allies against infectious diseases**

The discovery of dangerous, antibiotic-resistant bacterial strains worldwide is a growing concern [9]. This has prompted research into new antimicrobial agents, and medicinal plants are promising sources [10,11]. They contain alkaloids, flavonoids, terpenoids, and phenolic compounds, which provide diverse biological activities, including potent antibacterial properties [12]. These are effective against bacteria such as *Staphylococcus aureus*, *Micrococcus flavus*, *Bacillus cereus*, *B. subtilis*, *Salmonella enteritidis*, *Escherichia coli*, and *Pseudomonas aeruginosa*, among others [13,14,15]. Notably, the genus Artemisia has been of great interest due to its pharmacological properties, in particular antibacterial activity [16,17,18]. For example, extracts of *Artemisia absinthium* L., *Artemisia annua* L., *Artemisia monosperma* Delile, *Artemisia cina* Berg ex Poljakov, and *Artemisia argyi* H. Lév. & Vaniot, show antibacterial activity against both Gram-positive and Gram-negative bacteria. Gram-negative strains are the most susceptible [17,19,20]. *A. cina* is a promising antibacterial treatment due to its high flavonoid content. It can also biosynthesize other antibacterial compounds, which confer additional activity against fungi, insects, and parasites [17]. García Hernández and colleagues [21] found that the *n*-hexane extract and cinaguaiacin showed promising antibacterial activity against clinically relevant multidrug-resistant bacteria. Cinaguaiacin, specifically, inhibited all tested strains at low minimum inhibitory concentrations (MICs). This means a low amount of the compound was sufficient to inhibit bacterial growth. Furthermore, it caused membrane damage and interfered with DNA gyrase B in molecular docking studies. These results support the longstanding relevance of *A. cina* as an effective antimicrobial and underscore the need for new approaches to address antimicrobial resistance.

In the case of malaria, the parasite strains have developed resistance to many current drugs [22,23]. Therefore, developing new antimalarial treatments that act differently from current drugs is needed [24]. In this sense, Ochora and colleagues [25] designed an antimalarial drug by combining epirubicin and pelitinib drugs with the natural antimalarial compounds artemether and lumefantrine to analyze whether the synergy between these drugs and compounds improved the antimalarial therapy.

The in vitro findings showed that epirubicin, artemether, and lumefantrine acted synergistically. This synergy reduces the likelihood of developing drug-resistant parasites and increases overall therapeutic activity. This work demonstrates that drug repurposing for new purposes can accelerate the availability of treatments for persistent diseases.

Perfecting the efficacy of treatments in infectious diseases is an urgent public health challenge [26]. Developing new substances with antiviral, antibacterial, anticancer, and antioxidant properties is hard; however, advances in nanotechnology are providing a range of nanoparticle-based chemicals for medicinal applications [27,28]. In this regard, Mohammed et al. showed that selenium nanoparticles (Se-NPs) derived from *Aspergillus flavus* via microbial fermentation can be effective against various pathogens. These include *Salmonella typhimurium*, *Bacillus pumilus*, *Staphylococcus aureus*, *Clostridium sporogenes*, *Escherichia coli*, and *Bacillus subtilis* [29]. Se-NPs also exhibited anticancer activity against cell lines of pancreatic carcinoma, cervical cancer, and colorectal adenocarcinoma. The particles also show antiviral activity against herpes simplex virus type 1 and hepatitis A virus. These findings underscore that biocompatible and multifunctional nanomaterials align with global biomedical innovations.

**Natural products in regenerative medicine and advanced drug delivery**

The treatment for skin tears (STs) uses dressings that have a low incidence of friction, shear, and adhesion. The most used foams are high-cost [30]. Given the financial impact of ST treatments, more affordable options must be evaluated. One alternative is the *Copaifera multijuga* Hayne oleoresin (CMOR), extracted from the Copaifera tree, which has been used as a treatment due to its terpenes (copalic acid, β-caryophyllene, and α-copaene), which have anti-inflammatory, antibacterial, insecticidal, antifungal, and healing effects [31,32,33,34]. Cardinelli and colleagues found that skin tears treated with hydrogels containing 2% or 10% copaiba oleoresin healed rapidly, with improved granulation and epithelialization and no adverse events [35]. Therefore, Copaiba-based hydrogels, especially those with 2%, were considered a safe and effective candidate for routine clinical wound treatment.

The pentacyclic triterpenoids (TTs) represent a family of phytochemicals that includes terpenes and their hydroxylated or carboxylated derivatives. These TTs are considered antioxidants, anti-inflammatory agents, antivirals, hypoglycemic agents, and anticancer agents. This suggests they could be potential pharmacological agents [36,37,38,39,40]. However, the hydrophobicity, poor permeability, and low absorption hinder its bioavailability and efficacy. To enhance the efficacy and administration, non-toxic carriers such as nanoparticles and nanocolloids are used [41,42,43]. The best-known nanodelivery vehicles are liposomes; microscopic vesicles with a lipid bilayer that can incorporate lipophilic (fat-loving) drugs into the membrane bilayer [44,45]. Rugină et al. developed liposomal and nanostructured lipid carriers for TTs from *Betula pendula* bark [46]. These nanoformulations enhanced cellular uptake and increased apoptosis in melanoma and carcinoma cells. Its toxicity was lower than that of doxorubicin. The successful use of TTs in biodegradable nanocarriers demonstrates that delivery techniques can improve the therapeutic potential of natural compounds, especially for cancer therapy.

**Reviews**

Finalizing the Special Issue, two reviews were included. The first, by Mitea and colleagues [47], provides a comprehensive review of advances in molecular mechanisms and the use of combined chemotherapeutic therapies. It also covers novel formulation approaches such as nanoemulsions and mucoadhesive films made from plant-derived bioactive compounds for the treatment of oral squamous cell carcinoma (OSCC). The review defines a paradigm in which natural products serve not merely as chemotherapeutic adjuncts but as central elements of oncology strategies aimed at reducing toxicity and overcoming treatment resistance. In contrast, the second review analyzes advances in molecular function and recombinant expression of human collagen [48]. Specifically, it is mentioned that the plant expression system for collagen offers a wide range of host sources, low cost, and high security, which will play a significant role in large-scale collagen production. However, more research is needed to combine recombinant collagen with new biological materials and to analyze their effectiveness in biological applications.

**Conclusions**

The studies in this Special Issue highlight evidence of the achievements in the use of natural compounds, either as traditional phytochemicals, repurposed agents, or biologically synthesized nanomaterials, in biomedical science. They offer multifaceted mechanisms, favorable safety profiles, and versatility across diseases (cancer, malaria, microbial infections, and wound healing). Furthermore, the inclusion of proteomics, computational modeling, and nanotechnology is accelerating our ability to elucidate, optimize, and translate these compounds into clinically meaningful interventions.

As members of the editorial board, we highlight the author’s scientific rigor and innovative efforts. We warrant that this collection serves as a solid reference for further research into the therapeutic potential of sesquiterpene lactones and, more generally, all natural compounds, driving progress toward diverse, safer, and more effective medical therapies.
